# Positive Selection in *Bifidobacterium* Genes Drives Species-Specific Host–Bacteria Communication

**DOI:** 10.3389/fmicb.2019.02374

**Published:** 2019-10-15

**Authors:** Marina S. Dyachkova, Evgeny V. Chekalin, Valery N. Danilenko

**Affiliations:** Vavilov Institute of General Genetics, Russian Academy of Sciences, Moscow, Russia

**Keywords:** bifidobacteria, host–bacteria communication, adhesion, signal transduction, adaptive evolution, positive selection, Red Queen Hypothesis

## Abstract

Bifidobacteria are commensal microorganisms that inhabit a wide range of hosts, including insects, birds and mammals. The mechanisms responsible for the adaptation of bifidobacteria to various hosts during the evolutionary process remain poorly understood. Previously, we reported that the species-specific PFNA gene cluster is present in the genomes of various species of the *Bifidobacterium* genus. The cluster contains signal transduction and adhesion genes that are presumably involved in the communication between bifidobacteria and their hosts. The genes in the PFNA cluster show high sequence divergence between bifidobacterial species, which may be indicative of rapid evolution that drives species-specific adaptation to the host organism. We used the maximum likelihood approach to detect positive selection in the PFNA genes. We tested for both pervasive and episodic positive selection to identify codons that experienced adaptive evolution in all and individual branches of the *Bifidobacterium* phylogenetic tree, respectively. Our results provide evidence that episodic positive selection has played an important role in the divergence process and molecular evolution of sequences of the species-specific PFNA genes in most bifidobacterial species. Moreover, we found the signatures of pervasive positive selection in the molecular evolution of the *tgm* gene in all branches of the *Bifidobacterium* phylogenetic tree. These results are consistent with the suggested role of PFNA gene cluster in the process of specific adaptation of bifidobacterial species to various hosts.

## Introduction

Bifidobacteria are anaerobic bacteria, some of the most ancient representatives of the phylum *Actinobacteria* (Gao et al., 2006), which existed in the times when the Earth’s atmosphere contained little oxygen. Today, bifidobacteria are commensal microorganisms that inhabit a wide range of hosts, including insects, birds and mammals. The overview of bifidobacteria ecology suggests a strict association between bifidobacterial species and the animal niches that they occupy (Mattarelli et al., 2017). One possible explanation is that species-specific adaptation and long-term co-evolution led to the formation of this association. The mechanisms responsible for the adaptation of bifidobacteria to various hosts during the evolutionary process remain poorly understood.

Host colonization is accompanied by bidirectional host-commensal microbiota communication. Studies in the field of microbial endocrinology show that microorganisms, through their long coexistence with their hosts, evolved sensors for detecting signaling molecules produced by the latter (Hughes and Sperandio, 2008; Lesouhaitier et al., 2009; Freestone, 2013). Signal transduction systems allow microorganisms to respond adequately to the varying environmental conditions. One-component systems, represented by serine-threonine protein kinases (STPK), dominate signal transduction in prokaryotes (Ulrich et al., 2005). STPK and their associated phosphatases (STPP) play a key role in bacterial signal transduction by catalyzing reversible phosphorylation of substrates. First, kinases perceive external stimuli, then they undergo autophosphorylation after which they acquire the ability to phosphorylate substrates, a process that modulates their activity (Pereira et al., 2011). Also, in order for bacteria to colonize and interact with the host organism, it is important that they adhere to the mucus layer of the intestinal epithelium.

Previously, we identified and characterized the PFNA gene cluster (created from the initial letters of the three genes: ***p****kb2*, ***fn****3*, *aaa-****a****tp*) in the genomes of various species of bifidobacteria. The cluster contains signal transduction and adhesion genes and can potentially be involved in the communication between bifidobacteria and their hosts. The cluster consists of an evolutionary stable group of genes that were characterized by a high degree of interspecific sequence divergence: the *pkb2*, *fn3*, *aaa-atp*, *duf58*, and *tgm* genes. We confirmed the operon organization of the PFNA cluster (Nezametdinova et al., 2018). The *pkb2* gene encodes the STPK Pkb2. We have experimentally demonstrated the functionality of this kinase and identified proteins that were considered as possible Pkb2 substrates (Nezametdinova et al., 2014, 2018). The obtained data concerning the phosphorylation of Pkb2 substrates allowed us to assume that the kinase function is related to adhesion and communication of bifidobacteria with intestinal epithelial cells. In particular, among the experimentally confirmed phosphorylation substrates was the glutamine synthase GlnA1. Orthologs of the GlnA1 were discovered in the extracellular proteomes of several bifidobacteria strains (Vazquez-Gutierrez et al., 2017). This protein is classified as a moonlighting protein. In *B. animalis* subsp. *lactis*, it can bind to the human plasminogen and promote the adhesion of bifidobacteria to the host’s gut epithelium (Candela et al., 2007). In the pathogenic actinobacteria *Mycobacterium tuberculosis*, glutamine synthase can bind to plasminogen and fibronectin (Kainulainen and Korhonen, 2014). We found that the ATPase encoded by the *aaa-atp* gene is also one of the substrates for Pkb2 phosphorylation. The ATPase belongs to the MoxR family [subfamily MoxR Proper (MRP)]. The known functions of ATPases of the MRP subfamily are to modulate the activity of their substrates (Van Spanning et al., 1991; Toyama et al., 1998; Snider and Houry, 2006). Genetic surroundings of *mrp* genes in different species of microorganisms are often characterized by the presence of genes encoding a DUF58 domain-containing protein of unknown function and a transglutaminase downstream of the *mrp* gene (Wong and Houry, 2012). The same consistent arrangement of these three genes is found in the studied cluster PFNA (Nezametdinova et al., 2018). Transglutaminases are known to catalyze the post-translational modification of proteins by the formation of proteinase resistant isopeptide bonds (Griffin et al., 2002). The putative transglutaminase encoded by the *tgm* gene is a polytopic transmembrane protein, like many receptors, ion channels, and transporters, indicating potential involvement in environmental interactions. The *fn3* gene encodes a fibronectin type III (FN3) domain-containing protein which was experimentally shown to participate in the adhesion of bifidobacteria to human epithelial cells (Westermann, 2015).

In some cases, the genes responsible for communication with environmental factors were shown to have undergone rapid evolution (Voolstra et al., 2011; Singh et al., 2012). A special well-studied case of rapid evolution as a result of interaction with environmental factors is the effect described by the Red Queen Hypothesis (RQH). The RQH suggests that co-evolution of interacting species should drive molecular evolution through continual natural selection for adaptation and counter-adaptation (Van Valen, 1973, 1974; Stenseth and Smith, 1984). The divergence observed at some host-resistance (Hedrick, 1994; Obbard et al., 2006; Clark et al., 2007) and parasite-infectivity (Blanc et al., 2005; Mu et al., 2007; Barrett et al., 2009) genes is consistent with this. It was also experimentally demonstrated that the rate of molecular evolution in the parasite was far higher when both host and parasite co-evolved with each other than when the parasite evolved against a constant host genotype (Paterson et al., 2010). Antagonistic co-evolution is likely to be a major driver of evolutionary change within species. Development of the functional genetics of interactions and comparative analyses has also revealed that fast-evolving genes are commonly those at the interface of biotic interactions (Brockhurst et al., 2014). Although the RQH usually describes effects resulting from binary antagonistic interactions, it seems that the community context aspect also needs to be considered (Brockhurst et al., 2014). For instance, host’s gut carry a variety of pathogens, as well as commensals and beneficial microorganisms. While some immune pathways may be specific to particular pathogens, others may have interplay with different pathogens and beneficial symbionts. Adaptation of the host with respect to pathogens may thus impact commensals which, in turn, are forced to enter the evolutionary race. Another possible reason of entering the evolutionary race is the adaptation of bifidobacteria to their surrounding community (e.g., competition with other commensals and pathogens for niche occupation).

As mentioned above, the PFNA genes show high sequence divergence between species, which may be indicative of rapid evolution that have driven species-specific adaptation to the host organism. The aim of this work was to study the phenomenon of rapid evolution of the PFNA genes and to explain the high degree of sequence divergence between different species. We showed that positive selection have contributed to the rapid evolution of the PFNA genes.

## Results

### Phylogenetic Analysis

**FIGURE 1:**
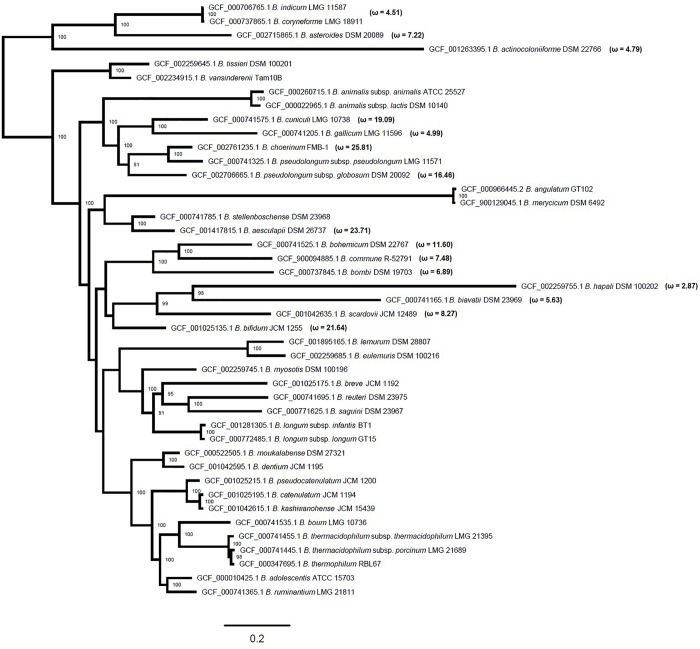
Maximum likelihood phylogenetic tree based on concatenated protein-coding sequences of the *pkb2*, *fn3*, *aaa-atp*, *duf58*, and *tgm* genes of 43 representatives of various species of the *Bifidobacterium* genus after 10,000 bootstrap replicates. Bootstrap values higher than 70 are marked next to the respective nodes showing a robust phylogenetic reconstruction. Values of ω are specified for a class of sites with ω > 1 in those branches of the tree, in which the evidence of episodic positive selection was obtained under strict conditions. The original branch lengths are presented; the scale bar corresponds to the value of two expected substitutions for ten nucleotide sites.

To confirm the co-evolution of PFNA genes, we used the MirrorTree method (Ochoa and Pazos, 2010). We calculated the pairwise Pearson correlation coefficients between the evolutionary distance matrices of phylogenetic trees based on multiple sequence alignments of the orthologous genes *pkb2*, *fn3*, *aaa-atp*, *duf58*, and *tgm* belonging to various bifidobacterial species. The values of the Pearson correlation coefficient were 0.800–0.978 (Supplementary Table S2) with the *P* < 0.000001. Pairwise comparison of phylogenetic trees showed high Pearson correlation coefficients, which confirms the co-evolution of the PFNA genes. This made it possible for us to use aligned concatenated sequences of the genes to build a phylogenetic tree. The use of sequences of concatenated genes as opposed to the use of sequences of individual genes increased the statistical power of the molecular evolution analysis and improved the accuracy of the obtained phylogenetic tree since a higher number of substitutions is analyzed. We constructed an unrooted *Bifidobacterium* phylogenetic tree based on the concatenated coding regions of the *pkb2*, *fn3*, *aaa-atp*, *duf58*, and *tgm* genes (Figure 1). The external nodes of the obtained phylogenetic tree were strongly supported by bootstrap values and, regardless of the slight differences, accurately reproduced the existing robust phylogenies of bifidobacteria (Lugli et al., 2014; Sun et al., 2015). The topology of the tree reproduced the following phylogroups of the *Bifidobacterium* genus: *B. asteroides*, *B. pseudolongum*, *B. longum, B. boum* groups (Sun et al., 2015), and *B. bifidum* group (Lugli et al., 2014, 2017, 2018). The evolutionary distances (*E*_D_) of the constructed phylogenetic tree for the *B. indicum* and *B. coryneforme* pair (*E*_D_ = 0.0089) and *B. catenulatum* and *B. kashiwanohense* pair (E_D_ = 0.02) were even shorter than for *B. animalis* subsp. *animalis* and *B. animalis* subsp. *lactis* pair (E_D_ = 0.0654) as well as *B. longum* subsp. *infantis* and *B. longum* subsp. *longum* pair (E_D_ = 0.0262) belonging to the same species. Thus, the pairs demonstrated high level of genetic relatedness. In contrast, the topology and evolutionary distance for the *B. pseudolongum* subsp. *pseudolongum* and *B. pseudolongum* subsp. *globosum* pair (*E*_D_ = 0.4054) indicated that there is a discrepancy between the conventional naming and the obtained tree. Our results are consistent with the reclassification proposed earlier (Lugli et al., 2014).

### Codon-Based Analyses of Positive Selection

Since recombination is known to produce false positive results (Anisimova et al., 2003), we screened the sequences for recombination events before running positive selection tests. We found no evidence of recombination in the studied sequences. Molecular evolution analysis was then performed using the maximum likelihood method. The method allows to detect evolutionary events of pervasive or episodic positive selection in the nucleotide sequences of protein-coding genes.

**FIGURE 2:**
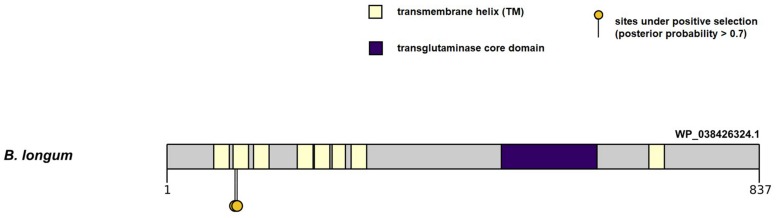
Localization of amino acid sites under pervasive positive selection in all branches of the *Bifidobacterium* phylogenetic tree in the primary structure of the WP_038426324.1 protein encoded by the *tgm* gene in *B. longum* subsp. *longum* GT15 genome. The figure shows the domain organization of the protein, as well as the localization of candidate sites with a PP value > 0.7.

First we tested the hypothesis for the presence of pervasive positive selection events in the molecular evolution of genes in the PFNA cluster. We obtained values of the log likelihood function for the site models M8 and M8a using CODEML program (Yang, 2007) and then we conducted likelihood ratio test (LRT) for the presence of sites under positive selection pressure (ω > 1) in all branches of the *Bifidobacterium* phylogenetic tree. The LRT value for the test was statistically significant (LRT = 175.19, *P* ≪ 0.01). Thus, *in silico* analysis showed that there is evidence for sites under the pressure of positive selection in all branches of the *Bifidobacterium* phylogenetic tree built on the basis of concatenated sequences of the PFNA genes. Then we identified the sites using the Bayes empirical Bayes (BEB) approach. Sites with a posterior probability (PP) > 0.7 were inferred to have evolved under positive selection. As a result, we detected two amino acid sites under pressure of positive selection in all branches of the phylogenetic tree: 97T (PP = 0.744, ω = 1.322 ± 0.309) and 100I (PP = 0.858, ω = 1.401 ± 0.248). Both candidate sites were located in the transmembrane (TM) domain of the protein encoded by the *tgm* gene of the PFNA cluster (Figure 2). The site coordinates are given for the sequence of the primary structure of the protein encoded by the *tgm* gene of the *B. longum* subsp. *longum* GT15 (WP_038426324.1).

Even though we detected events of pervasive positive selection in the molecular evolution of the *tgm* gene, we decided to test the hypothesis whether episodic positive selection also played a role in the molecular evolution of the PFNA genes. Episodic selection affecting individual sites in individual branches and clades of a phylogenetic tree is the most common case of positive selection. We obtained values of the log likelihood function for two branch-site models for the tested branches and clades of the *Bifidobacterium* phylogenetic tree and then we conducted LRT tests under strict and relaxed conditions. First, we applied the branch-site test 1, during which we tested the assumption of the presence of sites under positive selection (ω > 1)/under relaxed negative selection in the tested branch/set of branches (foreground branches) in comparison with other branches of *Bifidobacterium* phylogeny (M1a vs. A) (Zhang et al., 2005). The LRT values for a number of tested branches and clades of the phylogeny were statistically significant. In particular, we detected selection events in 20 test branches under strict conditions and in 27 test branches under relaxed conditions (Supplementary Table S3). The LRT values for the following branches and clades of the phylogenetic tree were statistically non-significant even when tested in relaxed conditions: *B. adolescentis*, *B. dentium*; *B. longum* subsp. *infantis*, *B. longum* subsp. *longum*; *B. moukalabense*; *B. reuteri*; *B. saguini*; *B. thermacidophilum* subsp. *porcinum*, *B. thermacidophilum* subsp. *thermacidophilum*, *B. thermophilum*; *B. vansinderenii*. Therefore, we obtained no evidence of positive selection/relaxed negative selection for these branches. Since test 1 was unable to distinguish between relaxation of selective constraint and positive selection (Zhang, 2004), we applied test 2 which was developed by the authors as a direct testing method for the detection of positive selection in the lineages of interest (Zhang et al., 2005). For the branches and clades of the phylogenetic tree that passed test 1, we tested the hypothesis that there are sites under pressure of positive selection (ω > 1) in the tested branch/set of branches compared to the other branches of phylogeny (A1 vs. A) (Zhang et al., 2005). We detected positive selection events in 15 out of 20 tested branches under strict conditions and in 26 out of 27 tested branches under relaxed conditions (Supplementary Table S4). Thus, *in silico* analysis proved the presence of independent positive selection events in the molecular evolution of the PFNA genes for most branches of the *Bifidobacterium* phylogenetic tree (26 out of 35 tested branches under relaxed conditions). It should be noted that in some cases, the species showed positive selection events in the molecular evolution of the PFNA genes correspond to the longest branches of the *Bifidobacterium* tree (e.g., *B. actinocoloniiforme*, *B. hapali*). The detection of higher rates of positive selection on these sequences could be due to an incomplete taxon sampling, impacting the ancestral sequence reconstruction and the computation of the different model parameters or due to *d*_*S*_ saturation. This problem is well known to lead sometimes to false positive results. The sites under the pressure of positive selection were then identified (Supplementary Table S5) as previously described and located in the primary structure of the proteins encoded by the genes *pkb2* (Supplementary Figure S1), *fn3* (Supplementary Figure S2), *aaa-atp* (Supplementary Figure S3), *duf58* (Supplementary Figure S4) and *tgm* (Supplementary Figure S5), which belong to representatives of various bifidobacterial species. Sites with PP values >0.95 were inferred to be the most reliable candidates for positive selection. To check the robustness of our results we used an additional approach. We found positive selection events in all tested branches and clades of the *Bifidobacterium* tree using MEME program (Murrell et al., 2012), which is consistent with our previous results. We found 662 positive selected sites with PP > 0.7 and 335 sites with PP > 0.95 among them. 48 sites matched those previously predicted using CODEML (Supplementary Table S5).

## Discussion

As the number of sequenced *Bifidobacterium* genomes available for analysis is increasing, genomic approaches have been pursued to understand the genetic and physiological traits involved in host colonization and other aspects of host–bacteria communication. Analysis of these genome sequences provided insights into the very intimate association of bifidobacteria with their hosts and the adaptation to their gastrointestinal habitat and led to the identification of a large number of genes with a potential role in these processes (Grimm et al., 2014; Zakharevich et al., 2019). Of particular interest among them is the species-specific PFNA cluster that we recently discovered which is perhaps a vivid example of the effect described by the RQH.

In this study, we performed *in silico* analysis to investigate the suggested rapid evolution of the PFNA genes in various bifidobacterial species. The genes showed high interspecific sequence divergence, which may be indicative of a rapid evolution that could contribute to species-specific adaptation to the host organism. We found signatures of pervasive positive selection in the molecular evolution of the *tgm* gene in all branches of the *Bifidobacterium* phylogenetic tree. Candidate sites are located in the TM domain of the encoded protein. Amino acid residues that form the secondary structure of TM domain generally experience pressure of negative selection because of the biophysical and functional limitations of the amino acid composition of transmembrane α-helices. However, in rare cases, the TM regions of proteins are affected by positive selection. The TM regions involved in binding to ligands, in particular, can experience rapid evolution, expanding the repertoire of binding ligands of the protein family (Spielman and Wilke, 2013). The putative transglutaminase encoded by the *tgm* gene is a polytopic transmembrane α-helical protein. A N-terminal region containing up to eight conjugated transmembrane α-helices (Figure 2) was found in the primary structure of the transglutaminase in various species of bifidobacteria. The region can contribute to the formation of the tertiary structure involved in ligand binding, which may explain the detected events of pervasive positive selection in the sequence of transmembrane α-helices of the protein.

**FIGURE 3:**
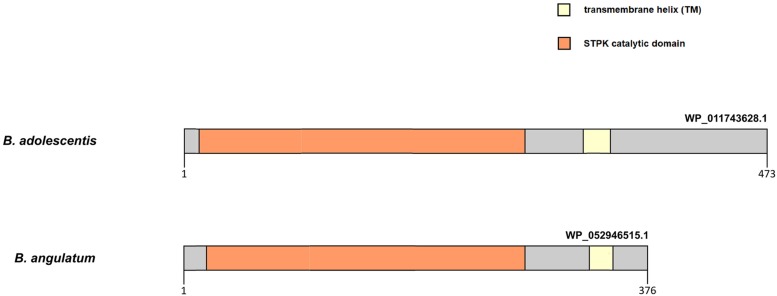
Difference in the primary structure of STPK Pkb2 from representatives of *B. angulatum* and *B. adolescentis* species belonging to the *B. adolescentis* phylogroup. Pkb2 contains a catalytic kinase domain, a transmembrane domain (TM), and a C-terminal signal region.

Our findings provide evidence that episodic positive selection has also played an important role in the divergence process and molecular evolution of sequences of the PFNA genes in most bifidobacterial species. We detected independent positive selection events in the PFNA genes sequences in various bifidobacterial species, which explains the high degree of interspecific divergence of the sequences. The tests that we performed support the notion of presence of groups of sites with a value of ω > 1 in the tested branches compared to the other branches of phylogeny. Therefore, positive selection in the molecular evolution of the PFNA genes turned out to be species-specific, affecting groups of sites in sequences corresponding to individual branches of phylogeny independently of others branches. The detected candidate sites (Supplementary Table S5) are located in various parts of the studied proteins, including annotated functional domains and transmembrane regions, as well as regions with no annotated functional domains. Episodic positive selection can drive rapid evolution in response to external factors. The adhesive properties of the protein encoded by the *fn3* gene may explain the phenomenon of rapid evolution in response to a species-specific change in the repertoire of adhesion substrates (Voolstra et al., 2011). The C-terminal region of the STPK Pkb2, which recognizes external stimuli, is highly variable in various species. The differences in the structure of this region, even in closely related species belonging to the same phylogroup (Figure 3), may indicate the species specificity of ligand binding. Unfortunately, we were not able to test the hypothesis of the presence of positive selection in these regions since, due to high divergence, they were almost completely excluded from the analysis after trimming the data (Supplementary Figure S6). As we mentioned before, the putative transglutaminase Tgm also appears to be involved in signal transduction. The episodic positive selection in molecular evolution of *pkb2* and *tgm* genes may have driven the implementation of the species-specific signaling mechanisms. The rapid evolution of at least one of the PFNA genes under the influence of external factors could have driven the rapid evolution of the co-evolving genes, which would have led to the high degree of interspecific divergence that we observed. In particular, the discovered positive selection in the *aaa-atp* gene may be the result of positive selection in the *pkb2* gene. The products of these genes physically interact with each other and hence were forced to co-adapt their structure in the process of co-evolution.

The LRT value for the *B. breve* branch was statistically significant in test 1 under strict conditions (Supplementary Table S3) (ω = 1) and statistically non-significant in test 2 even under relaxed conditions (Supplementary Table S4) which indicates possible relaxation of negative constraint during the molecular evolution of sequences. The PFNA cluster of *B. breve* contains a fusion gene that is a result of a combination of the *fn3* and *aaa-atp* genes. The evolutionary event resulting in such fusion must have occurred relatively recently since even the most closely related species of bifidobacteria contain in their genomes the sequences of the individual *fn3* and *aaa-atp* genes. We know that unlike evolutionarily older proteins, young proteins tend to weaken negative selection constraint, which makes them a subject to rapid evolution (Domazet-Loso and Tautz, 2003; Daubin and Ochman, 2004; Albà and Castresana, 2005, 2007; García-Vallvé et al., 2005; Wolf et al., 2009).

The LRT values for a number of branches and clades of the *Bifidobacterium* phylogenetic tree were statistically non-significant even when we tested them in relaxed conditions. Thus, we did not find any evidence of positive selection occurring in these branches. On the other hand, this can also be explained by a decrease in the statistical power of the tests due to relatively low branch lengths in some cases or an inevitable increase in false-negative error due to the use of the multiple testing correction.

Since the candidate sites for positive selection may be structurally or functionally significant, they can help expand the structural and functional protein annotation. The annotation of the proteins encoded by the PFNA genes is an important task since both the structure and function of these proteins remain poorly understood.

The diversity of animal niches colonized by bifidobacteria and the fact that even closely related species may inhabit guts of different species with different physiological and biochemical characteristics indicate that bifidobacteria having experienced the need to adapt to new conditions underwent divergent evolution. At the sequence level, this could be provided by positive selection that drives rapid changes in the structure of proteins. At the same time, the intimate association of bifidobacterial species with their hosts indicate that it was formed as a result of long-term co-evolution process. Our findings provide evidence for positive selection affecting genes potentially involved in host–bacteria communication, which is tempting to interpret in the context of the RQH. In particular, commensal microbiota, like pathogens, are under constant pressure of the immune response factors. The host’s immune system aims to eliminate pathogens, but beneficial microorganisms can also get caught in crossfire. Thus, commensal bacteria may also be forced into the evolutionary arms race. Long-term co-evolution of interacting species could drive the molecular evolution of genes contributing to this interaction.

## Materials and Methods

### Sequences

The sequences of the coding regions of the *pkb2*, *fn3*, *aaa-atp*, *duf58*, and *tgm* genes used in the analyses were retrieved from RefSeq^1^. We used gene sequences from the genomes of representatives of 43 different species and subspecies of bifidobacteria (Supplementary Table S1).

### Interspecific Alignments of the Sequences

The multiple codon alignments of the 43 nucleotide sequences of the *pkb2* (Supplementary Data Sheet S2), *fn3* (Supplementary Data Sheet S3), *aaa-atp* (Supplementary Data Sheet S4), *duf58* (Supplementary Data Sheet S5), and *tgm* (Supplementary Data Sheet S6) genes were performed independently in the ClustalW program (Thompson et al., 1994) implemented in the MEGA software v.7.0.14 (Kumar et al., 2016) using default settings. To eliminate poorly aligned positions and divergent regions, Gblocks v.0.91b (Castresana, 2000; Talavera and Castresana, 2007) was used with the default parameters (Supplementary Figure S6) and the resulting fragments were concatenated (Supplementary Data Sheet S7).

### Phylogenetic Analysis

Before phylogenetic analysis, the best-fit partitioning scheme and the substitution models for each partition were determined using PartitionFinder v.1.1.1 (Lanfear et al., 2016) under the Akaike (AIC), the corrected Akaike (AICc), and the Bayesian (BIC) information criteria. We determined the best-fit model of molecular evolution to be GTR + Γ + I. The maximum likelihood unrooted tree was generated using RAxML v.8.2.7 (Stamatakis, 2014) with 10,000 bootstrap replicates. The assignment of an outgroup for the tree construction was not possible since orthologs of the PFNA genes were not found in genomes of other organisms. The co-evolution of the PFNA genes was studied using the MirrorTree Server^2^ (Ochoa and Pazos, 2010).

### Codon-Based Analyses of Positive Selection

The analysis of possible recombination events was performed using GARD program^3^ (Kosakovsky Pond et al., 2006).

To examine the impact of positive selection on the PFNA genes, statistical tests for evaluating adaptive evolution were conducted using CODEML program as implemented in PAML software package v.4.8 (Yang, 2007). Site models (M8, M8a) and branch-site models (M1a, A, A1) were executed to detect the possibility of positive selection acting at a particular sites along all lineages of the phylogenetic tree or particular lineages (known as foreground branches), respectively (Yang and Nielsen, 2002). Opposing models were compared (M8 vs. M8a, M1a vs. A, and A1 vs. A) and LRTs were applied to select the ones that best fitted the data. The number of degrees of freedom (df) was calculated as the difference between the number of free parameters of compared models. Positive selection was inferred when codons with *d*_N_/*d*_S_ ratio (ω) > 1 were identified.

The best-fit value of the CodonFreq parameter was determined in the M1 model (*B. longum* was assigned as a foreground clade) under AIC, AICc, and BIC information criteria. The parameter specifies the equilibrium codon frequencies in codon substitution model. We determined the best-fit value of the parameter to be CodonFreq = 7. To calculate the correct branch lengths of the phylogenetic tree for the codon-based analysis of positive selection, the model M0 was used (fix_blength = 0), and then the branch lengths were fixed for all tests (fix_blength = 2).

Branch-site tests for the presence of positive selection/relaxed negative selection were performed independently for 35 individual branches and clades of the *Bifidobacterium* phylogenetic tree under strict conditions (χ^2^-distribution of LRT statistics, *P* < 0.01) and relaxed conditions (50:50 mixture distribution of the χ^2^-distribution and a point mass of zero, *P* < 0.05). The relaxed conditions were used to reduce the probability of a false-negative error since the test is conservative under the strict conditions (Zhang et al., 2005). To reduce the probability of a false-positive error as a result of multiple testing, the Bonferroni correction (strict conditions) and the Benjamini-Hochberg procedure (relaxed conditions) were used (Anisimova and Yang, 2007).

Since low values of the branch lengths lead to a significant weakening of the statistical power of the tests, short branches were tested as part of the following clades of the phylogenetic tree: *B. angulatum*, *B. merycicum*; *B. animalis* subsp. *animalis*, *B. animalis* subsp. *lactis*; *B. catenulatum*, *B. kashiwanohense*, *B. pseudocatenulatum*; *B. coryneforme*, *B. indicum*; *B. longum* subsp. *infantis*, *B. longum* subsp. *longum*; *B. thermacidophilum* subsp. *porcinum*, *B. thermacidophilum* subsp. *thermacidophilum*, *B. thermophilum*.

When the LRT was significant, the BEB method was used to identify codons that were likely to evolve under positive selection based on a PP thresholds of 0.7 and 0.95. Localization of sites under the pressure of positive selection in the primary structure of proteins was visualized in the IBS program v.1.0.

To check the robustness of our results we conducted additional positive selection tests using MEME program as implemented in HyPhy software package v.2.2.4 (Murrell et al., 2012).

## Data Availability Statement

All datasets generated for this study are included in the manuscript/Supplementary Files.

## Author Contributions

MD contributed to the design and implementation of the research, analysis of the results and to the preparation, and creation and presentation of the published work. EC contributed to the provision of computing resources and assisted in the analysis. VD supervised the project. All authors provided critical feedback and helped to shape the research, analysis, and manuscript.

## Conflict of Interest

The authors declare that the research was conducted in the absence of any commercial or financial relationships that could be construed as a potential conflict of interest.

## Footnotes

1 https://www.ncbi.nlm.nih.gov/refseq/

2 http://csbg.cnb.csic.es/mtserver/

3 http://www.datamonkey.org/gard

## Supplementary Material

FIGURE S1 Localization of amino acid sites under episodic positive selection in the primary structure of the protein encoded by the *pkb2* gene of various species of bifidobacteria. The figure shows the domain organization of the protein, as well as the localization of candidate sites with a value of PP = 0.7–0.95 and PP > 0.95.

FIGURE S2 Localization of amino acid sites under episodic positive selection in the primary structure of the protein encoded by the *fn3* gene of various species of bifidobacteria. The figure shows the domain organization of the protein, as well as the localization of candidate sites with a value of PP = 0.7–0.95 and PP > 0.95.

FIGURE S3 Localization of amino acid sites under episodic positive selection in the primary structure of the protein encoded by the *aaa-atp* gene of various species of bifidobacteria. The figure shows the domain organization of the protein, as well as the localization of candidate sites with a value of PP = 0.7–0.95 and PP > 0.95.

FIGURE S4 Localization of amino acid sites under episodic positive selection in the primary structure of the protein encoded by the *duf58* gene of various species of bifidobacteria. The figure shows the domain organization of the protein, as well as the localization of candidate sites with a value of PP = 0.7–0.95 and PP > 0.95.

FIGURE S5 Localization of amino acid sites under episodic positive selection in the primary structure of the protein encoded by the *tgm* gene of various species of bifidobacteria. The figure shows the domain organization of the protein, as well as the localization of candidate sites with a value of PP = 0.7–0.95 and PP > 0.95.

FIGURE S6 Localization of selected blocks after treatment with Gblocks (blue color) in the *pkb2* (BLGT_–_RS02820), *fn3* (BLGT_–_RS02815), *aaa-atp* (BLGT_–_RS02810), *duf58* (BLGT_–_RS02805), and *tgm* (BLGT_–_RS02800) sequences of *B. longum* subsp. *longum* GT15.

TABLE S1 The list of genomic sequences used in this study to analyze the molecular evolution of the PFNA genes of various bifidobacterial species.

TABLE S2 The calculated pairwise Pearson correlation coefficients between the evolutionary distance matrices of phylogenetic trees based on multiple sequence alignments of the orthologous genes *pkb2*, *fn3*, *aaa-atp*, *duf58*, and *tgm* belonging to various bifidobacterial species.

TABLE S3 Values of the LRTs that were applied to evaluate the presence of episodic positive selection / relaxed negative selection in the sequences of the PFNA genes. The LRTs was performed for different branches of the phylogenetic tree of bifidobacteria in strict and relaxed conditions. The corresponding *p*-values are given. The result is statistically significant when *P* < α.

TABLE S4 Values of the LRT that were applied to evaluate the presence of episodic positive selection in the sequences of the PFNA genes. The LRTs was performed in strict and relaxed conditions for the branches of the phylogenetic tree of bifidobacteria that passed test 1. The corresponding *p*-values are given. The result is statistically significant when *P* < α. The ω values are specified for a group of sites of the foreground branch with a value of ω > 1.

TABLE S5 Candidate amino acid sites under episodic positive selection in various tested branches of the phylogenetic tree based on concatenated sequences of the PFNA genes (PP > 0.7). The sites with PP > 0.95 are marked with an asterisk. The most reliable candidates for positive selection verified with an additional approach are highlighted in black.

DATA SHEET S2 MSA of the *pkb2* gene before treatment with Gblocks.

DATA SHEET S3 MSA of the *fn3* gene before treatment with Gblocks.

DATA SHEET S4 MSA of the *aaa-atp* gene before treatment with Gblocks.

DATA SHEET S5 MSA of the *duf58* gene before treatment with Gblocks.

DATA SHEET S6 MSA of the *tgm* gene before treatment with Gblocks.

DATA SHEET S7 MSA of the concatenated PFNA genes after treatment with Gblocks.
