# Supplementary material for: Positive Selection in Bifidobacterium Genes Drives Species-Specific Host–Bacteria Communication
Source: Front Microbiol. 2019 Oct 15;10:2374. doi: 10.3389/fmicb.2019.02374 (PMC6803598; doi:10.3389/fmicb.2019.02374)
Supplement: FIGURE S1 — Localization of amino acid sites under episodic positive selection in the primary structure of the protein encoded by the pkb2 gene of various species of bifidobacteria. The figure shows the domain organization of the protein, as well as the localization of candidate sites with a value of PP = 0.7–0.95 and PP > 0.95. [file Data_Sheet_1.PDF]

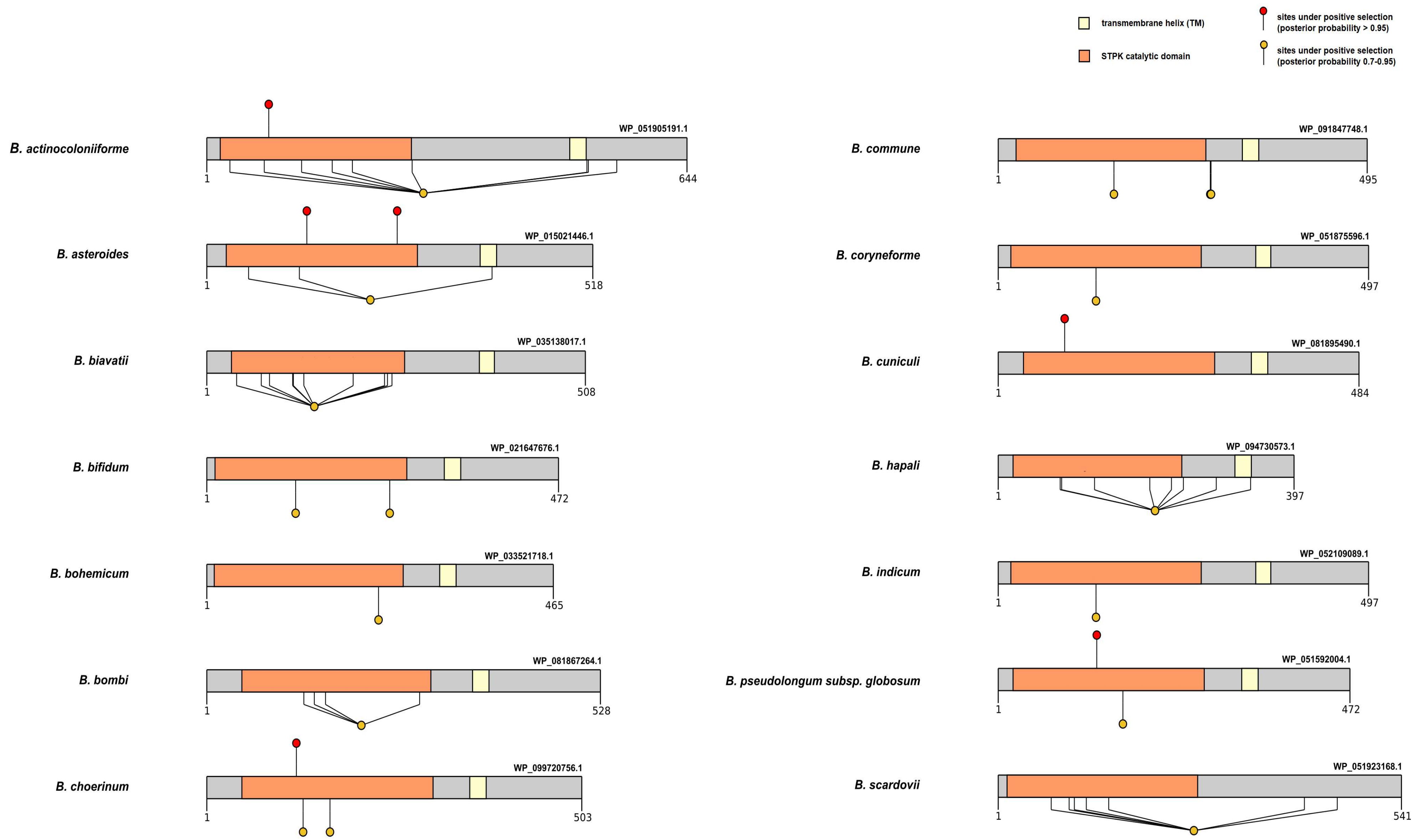

Figure S1. Localization of amino acid sites under episodic positive selection in the primary structure of the protein encoded by the *pkb2* gene of various species of bifidobacteria. The figure shows the domain organization of the protein, as well as the localization of candidate sites with a value of PP = 0.7-0.95 and PP > 0.95

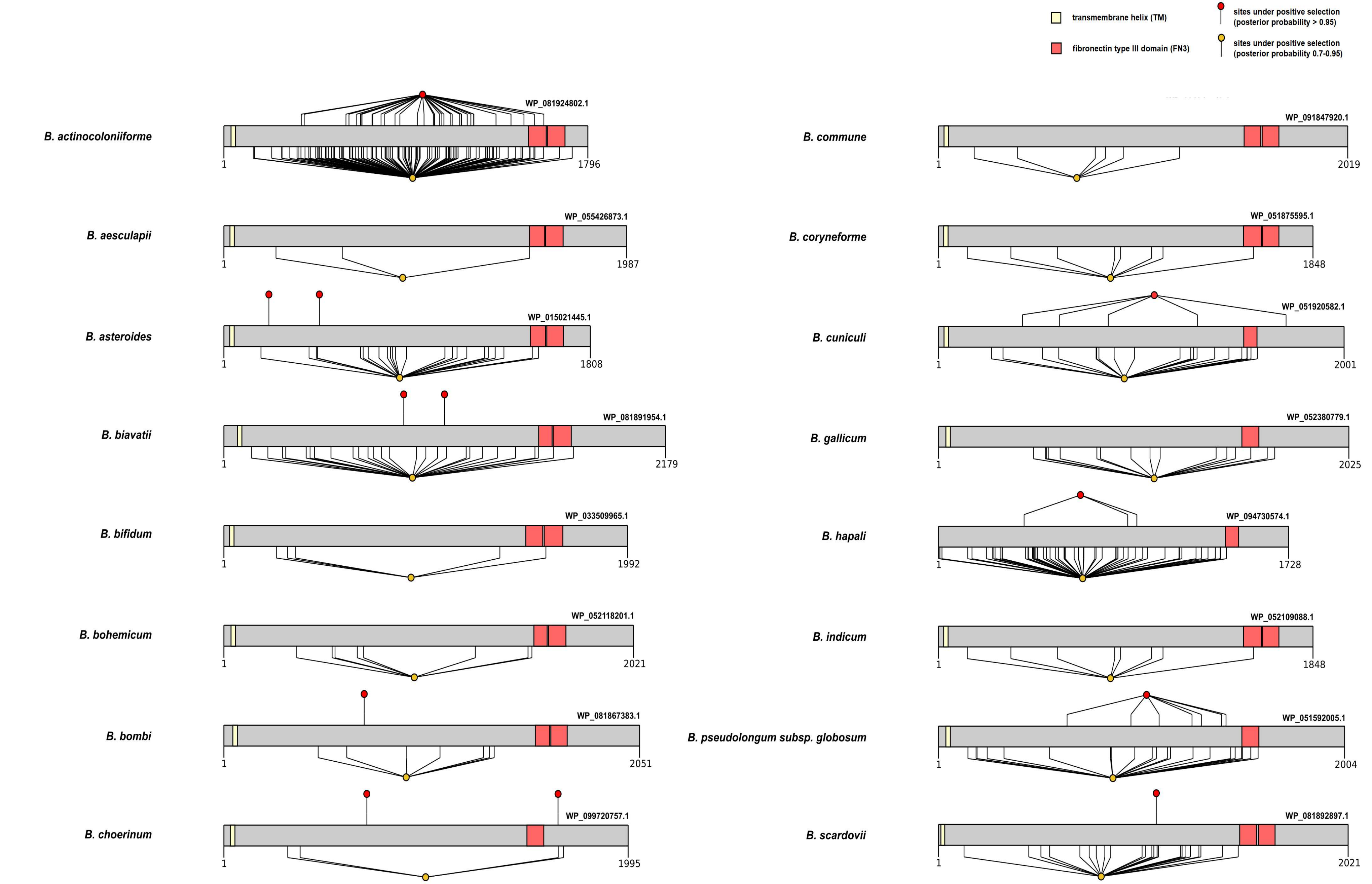

Figure S2. Localization of amino acid sites under episodic positive selection in the primary structure of the protein encoded by the *fn3* gene of various species of bifidobacteria. The figure shows the domain organization of the protein, as well as the localization of candidate sites with a value of PP = 0.7-0.95 and PP > 0.95.

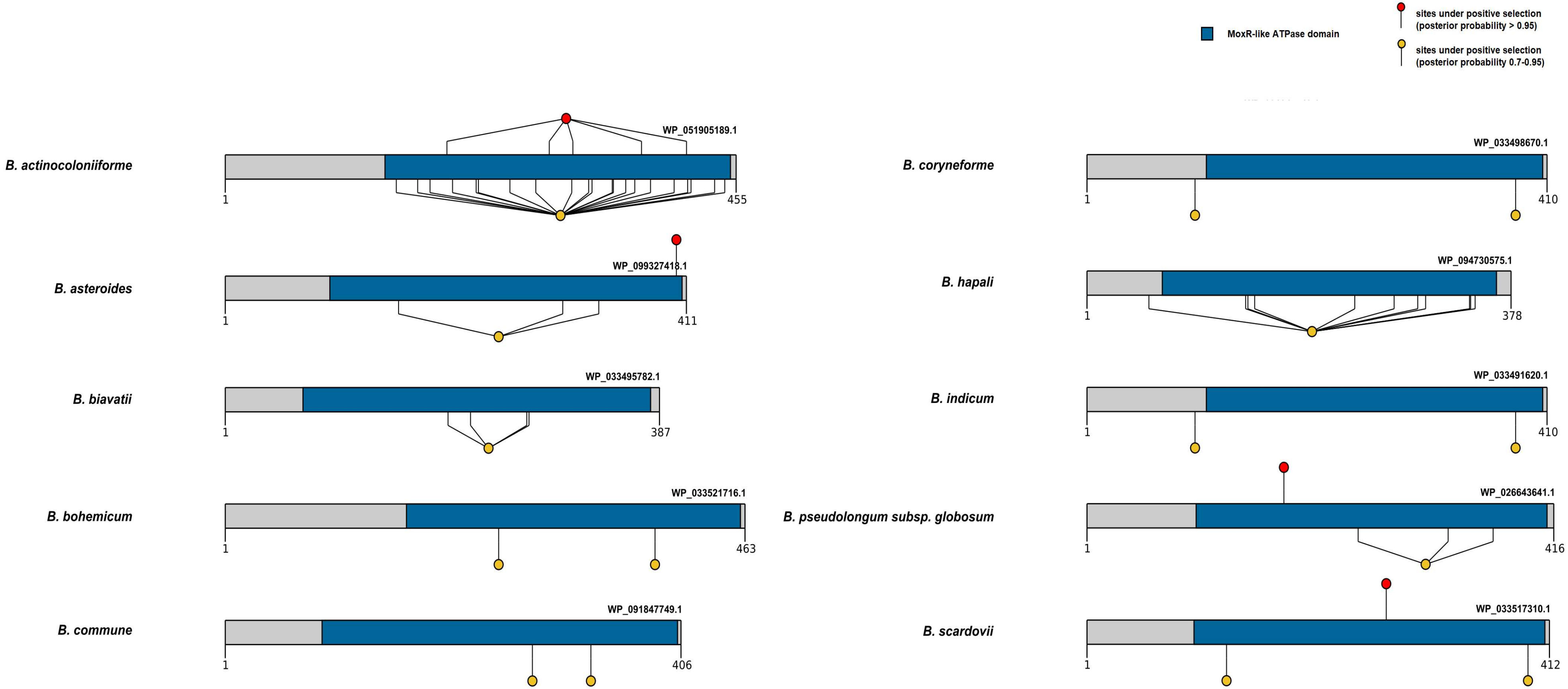

Figure S3. Localization of amino acid sites under episodic positive selection in the primary structure of the protein encoded by the *aaa-atp* gene of various species of bifidobacteria. The figure shows the domain organization of the protein, as well as the localization of candidate sites with a value of PP = 0.7-0.95 and PP > 0.95.

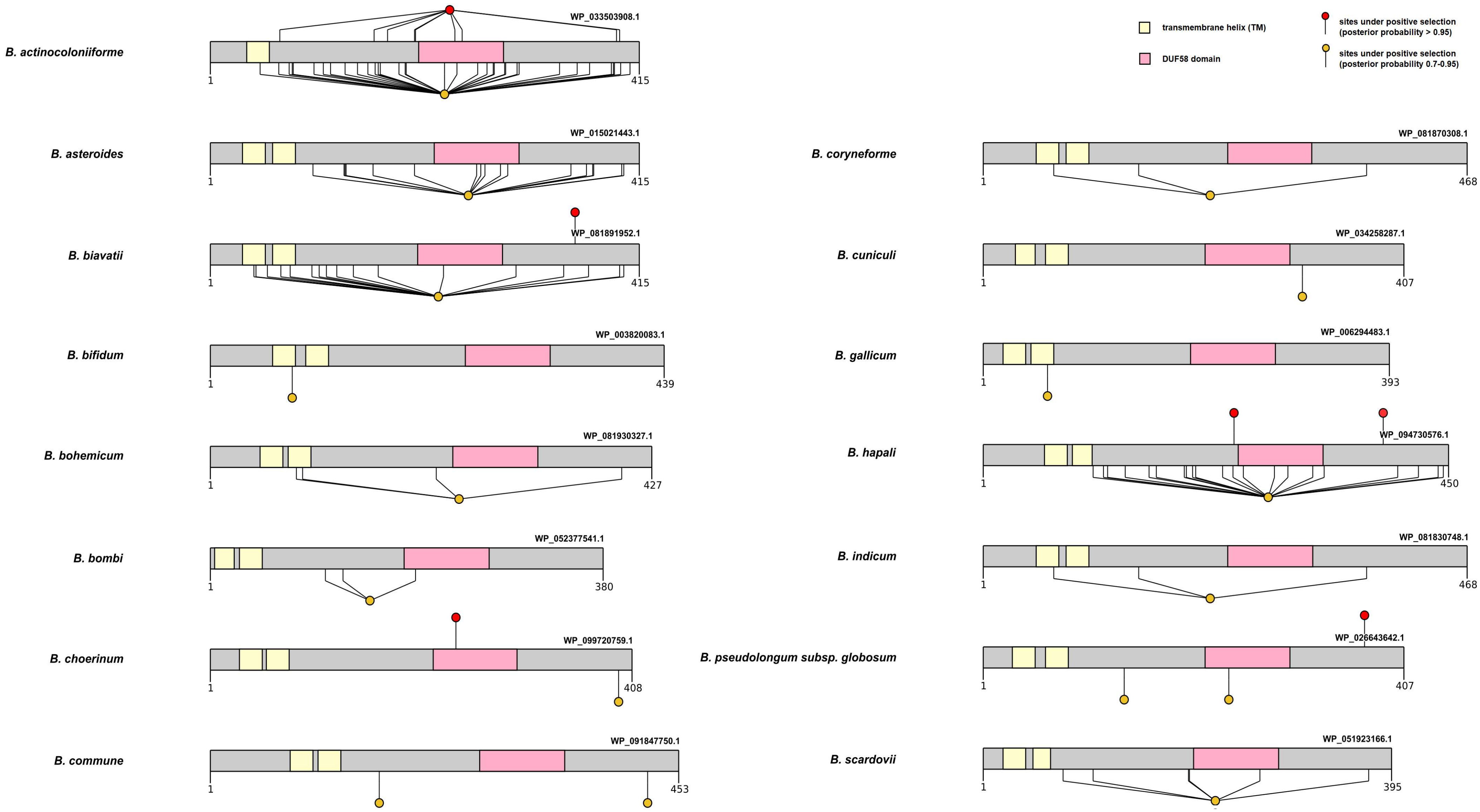

Figure S4. Localization of amino acid sites under episodic positive selection in the primary structure of the protein encoded by the *duf58* gene of various species of bifidobacteria. The figure shows the domain organization of the protein, as well as the localization of candidate sites with a value of PP = 0.7-0.95 and PP > 0.95.

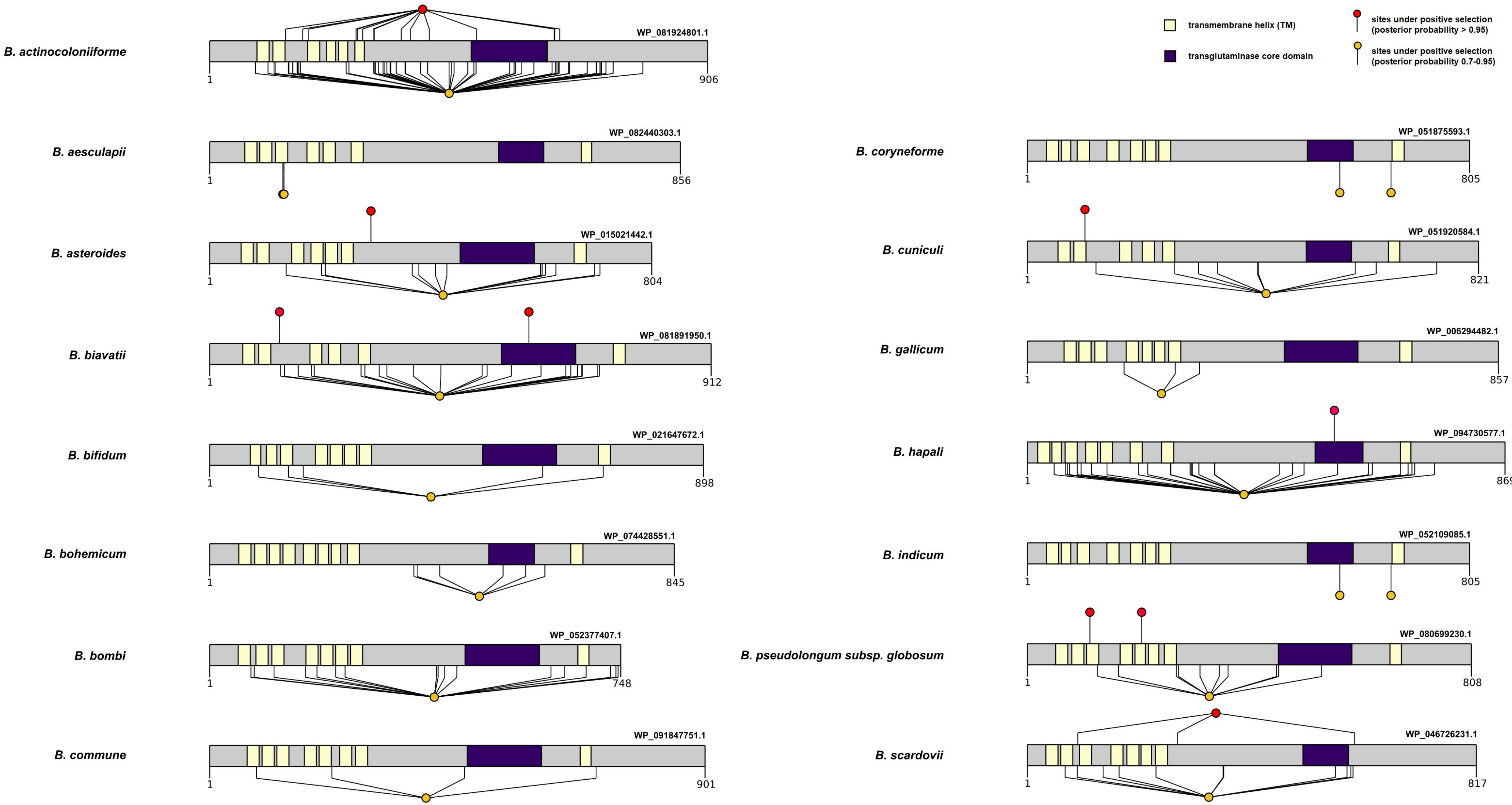

Figure S5. Localization of amino acid sites under episodic positive selection in the primary structure of the protein encoded by the *tgm* gene of various species of bifidobacteria. The figure shows the domain organization of the protein, as well as the localization of candidate sites with a value of PP = 0.7-0.95 and PP > 0.95.

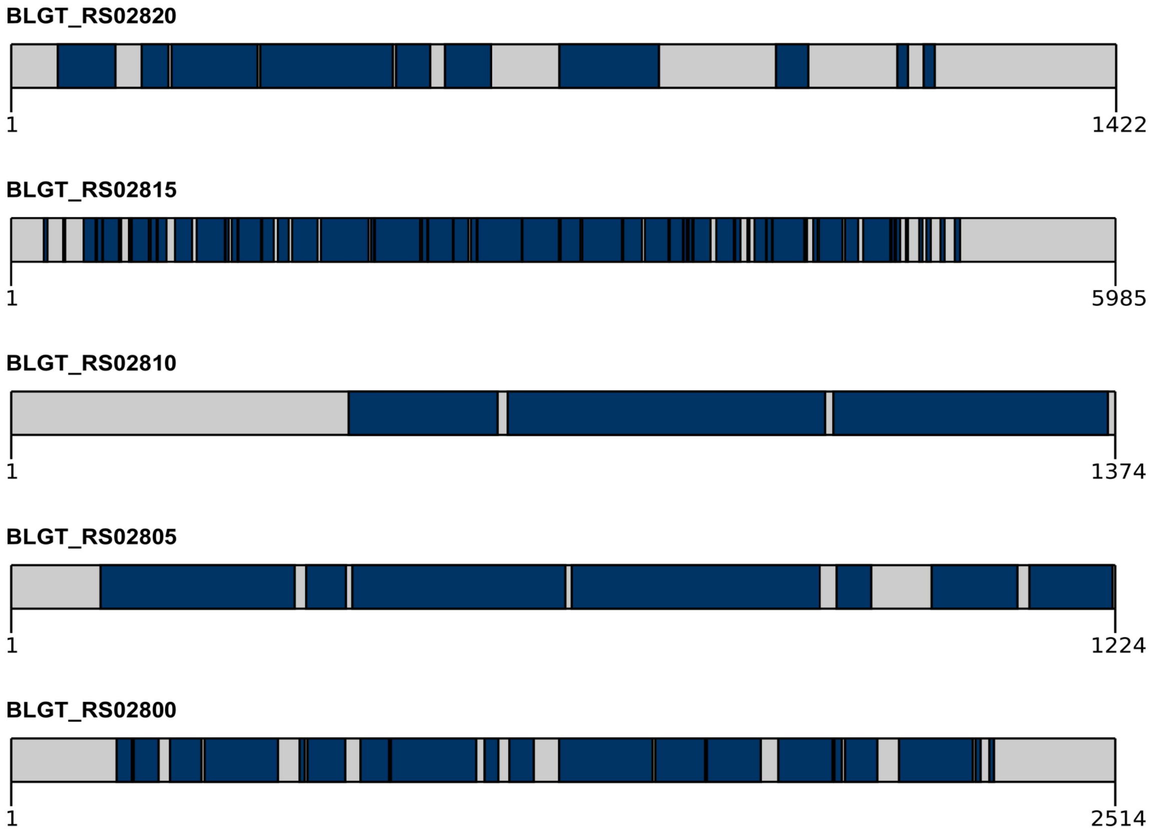

Figure S6. Localization of selected blocks after treatment with Gblocks (blue color) in the *pkb2* (BLGT_RS02820), *fn3* (BLGT_RS02815), *aaa-atp* (BLGT_RS02810), *duf58* (BLGT_RS02805), and *tgm* (BLGT_RS02800) sequences of *B. longum* subsp. *longum* GT15.
