# Supplementary material for: Positive Selection in Bifidobacterium Genes Drives Species-Specific Host–Bacteria Communication
Source: Front Microbiol. 2019 Oct 15;10:2374. doi: 10.3389/fmicb.2019.02374 (PMC6803598; doi:10.3389/fmicb.2019.02374)
Supplement: TABLE S1 — The list of genomic sequences used in this study to analyze the molecular evolution of the PFNA genes of various bifidobacterial species. [file Data_Sheet_8.PDF]

Table S1. The list of genomic sequences used in this study to analyze the molecular evolution of the PFNA genes of various bifidobacterial species.

species	subspecies	strain	assembly accession	assembly level	source of isolation
Bifidobacterium actinocoloniiforme		DSM 22766	GCF_001263395.1	complete	bumblebee gut
Bifidobacterium adolescentis		ATCC 15703	GCF_000010425.1	complete	adult intestine
Bifidobacterium aesculapii		DSM 26737	GCF_001417815.1	draft	baby common marmoset feces
Bifidobacterium angulatum		GT102	GCF_000966445.2	complete	adult feces
Bifidobacterium animalis	*animalis*	ATCC 25527	GCF_000260715.1	complete	rat faeces
Bifidobacterium animalis	*lactis*	DSM 10140	GCF_000022965.1	complete	fermented milk
Bifidobacterium asteroides		DSM 20089	GCF_002715865.1	complete	honeybee intestine
Bifidobacterium biavatii		DSM 23969	GCF_000741165.1	draft	red-handed tamarin feces
Bifidobacterium bifidum		JCM 1255	GCF_001025135.1	complete	infant feces
Bifidobacterium bohemicum		DSM 22767	GCF_000741525.1	draft	bumblebee gut
Bifidobacterium bombi		DSM 19703	GCF_000737845.1	draft	bumblebee gut
Bifidobacterium boum		LMG 10736	GCF_000741535.1	draft	bovine rumen
Bifidobacterium breve		JCM 1192	GCF_001025175.1	complete	infant intestine
Bifidobacterium catenulatum		JCM 1194	GCF_001025195.1	complete	adult intestine
Bifidobacterium choerinum		FMB-1	GCF_002761235.1	complete	bovine rumen
Bifidobacterium commune		R-52791	GCF_900094885.1	draft	bumblebee gut
Bifidobacterium coryneforme		LMG 18911	GCF_000737865.1	complete	honeybee hindgut
Bifidobacterium cuniculi		LMG 10738	GCF_000741575.1	draft	rabbit faeces
Bifidobacterium dentium		JCM 1195	GCF_001042595.1	complete	human dental caries
Bifidobacterium eulemuris		DSM 100216	GCF_002259685.1	draft	black lemur faeces
Bifidobacterium gallicum		LMG 11596	GCF_000741205.1	draft	adult intestine
Bifidobacterium hapali		DSM 100202	GCF_002259755.1	draft	baby common marmoset faeces
Bifidobacterium indicum		LMG 11587	GCF_000706765.1	complete	honeybee intestine
Bifidobacterium kashiwanohense		JCM 15439	GCF_001042615.1	complete	infant feaces
Bifidobacterium lemurum		DSM 28807	GCF_001895165.1	draft	ring-tailed lemur feaces
Bifidobacterium longum	*infantis*	BT1	GCF_001281305.1	complete	infant feces
Bifidobacterium longum	*longum*	GT15	GCF_000772485.1	complete	adult feces
Bifidobacterium merycicum		DSM 6492	GCF_900129045.1	draft	bovine rumen
Bifidobacterium moukalabense		DSM 27321	GCF_000522505.1	draft	wild western lowland gorilla feaces
Bifidobacterium myosotis		DSM 100196	GCF_002259745.1	draft	baby common marmoset feaces
Bifidobacterium pseudocatenulatum		JCM 1200	GCF_001025215.1	complete	infant faeces

Bifidobacterium pseudolongum	*globosum*	DSM 20092	GCF_002706665.1	complete	bovine rumen
Bifidobacterium pseudolongum	*pseudolongum*	LMG 11571	GCF_000741325.1	draft	swine faeces
Bifidobacterium reuteri		DSM 23975	GCF_000741695.1	draft	common marmoset feaces
Bifidobacterium ruminantium		LMG 21811	GCF_000741365.1	draft	bovine rumen
Bifidobacterium saguini		DSM 23967	GCF_000771625.1	draft	red-handed tamarin feaces
Bifidobacterium scardovii		JCM 12489	GCF_001042635.1	complete	human blood
Bifidobacterium stellenboschense		DSM 23968	GCF_000741785.1	draft	red-handed tamarin feaces
Bifidobacterium thermacidophilum	*porcinum*	LMG 21689	GCF_000741445.1	draft	piglet faeces
Bifidobacterium thermacidophilum	*thermacidophilum*	LMG 21395	GCF_000741455.1	draft	anaerobic digester
Bifidobacterium thermophilum		RBL67	GCF_000347695.1	complete	infant feces
Bifidobacterium tissieri		DSM 100201	GCF_002259645.1	draft	baby common marmoset feaces
Bifidobacterium vansinderenii		Tam10B	GCF_002234915.1	draft	emperor tamarin feaces

Table S2. The calculated pairwise Pearson correlation coefficients between the evolutionary distance matrices of phylogenetic trees based on multiple sequence alignments of the orthologous genes *pkb2*, *fn3*, *aaa-atp*, *duf58* and *tgm* belonging to various bifidobacterial species.

	pkb2	***fn3***	***aaa-atp***	***duf58***	***tgm***
pkb2	1				
fn3	0.800	1			
aaa-atp	0.814	0.966	1		
duf58	0.814	0.955	0.967	1	
tgm	0.813	0.976	0.978	0.976	1

Table S3. Values of the LRTs that were applied to evaluate the presence of episodic positive selection / relaxed negative selection in the sequences of the PFNA genes. The LRTs was performed for different branches of the phylogenetic tree of bifidobacteria in strict and relaxed conditions. The corresponding p-values are given. The result is statistically significant when P < α.

**foreground branch / clade**	**lnL value (M1a model)**	**lnL value (A model)**	**LRT value**	**P-value (strict conditions, α = 2.86E-004)**	**P-value (relaxed conditions, α = 0.05)**
B. actinocoloniiforme	-212064.17	-211851.62	425.10	0	0
B. adolescentis	-212064.17	-212064.17	0	1	1
B. aesculapii	-212064.17	-212052.63	23.08	9.74E-006	8.5225E-006
B. angulatum, *B. merycicum*	-212064.17	-211963.15	202.03	0	0
B. animalis subsp. *animalis*, *B. animalis* subsp. *lactis*	-212064.17	-212000.55	127.23	0	0
B. asteroides	-212064.17	-211985.51	157.31	0	0
B. biavatii	-212064.17	-211976.88	174.58	0	0
B. bifidum	-212064.17	-212047.66	33.01	7.00E-008	6.805556E-008
B. bohemicum	-212064.17	-212044.93	38.48	0	0
B. bombi	-212064.17	-212027.85	72.63	0	0
B. boum	-212064.17	-212060.78	6.78	3.37E-002	2.27E-002
B. breve	-212064.17	-212050.48	27.38	1.13E-006	1.040789E-006
B. catenulatum, *B. kashiwanohense*, *B. pseudocatenulatum*	-212064.17	-212060.94	6.46	3.95E-002	2.56E-002
B. choerinum	-212064.17	-212042.51	43.32	0	0
B. commune	-212064.17	-212047.15	34.03	4.00E-008	4.117647E-008
B. coryneforme, *B. indicum*	-212064.17	-212022.76	82.82	0	0
B. cuniculi	-212064.17	-212007.55	113.23	0	0
B. dentium	-212064.17	-212064.17	0	1	1
B. eulemuris	-212064.17	-212015.17	98.00	0	0

B. gallicum	-212064.17	-212041.48	45.37	0	0
B. hapali	-212064.17	-211948.99	230.36	0	0
B. lemurum	-212064.17	-212016.75	94.84	0	0
B. longum subsp. *infantis*,<br>*B. longum* subsp. *longum*	-212064.17	-212064.17	0	1	1
B. moukalabense	-212064.17	-212064.17	0	1	1
B. myosotis	-212064.17	-212058.82	10.69	4.76E-003	3.63E-003
B. pseudolongum subsp. *globosum*	-212064.17	-211994.42	139.50	0	0
B. pseudolongum subsp. *pseudolongum*	-212064.17	-212059.50	9.33	9.43E-003	6.88E-003
B. reuteri	-212064.17	-212062.75	2.84	2.42E-001	1.40E-001
B. ruminantium	-212064.17	-212059.56	9.22	9.94E-003	6.96E-003
B. saguini	-212064.17	-212064.17	0	1	1
B. scardovii	-212064.17	-211991.13	146.07	0	0
B. stellenboschense	-212064.17	-212056.48	15.37	4.59E-004	3.83E-004
B. thermacidophilum subsp. *porcinum*,<br>*B. thermacidophilum* subsp.<br>*thermacidophilum*,<br>*B. thermophilum*	-212064.17	-212062.36	3.62	1.64E-001	9.90E-002
B. tissieri	-212064.17	-212058.77	10.79	4.53E-003	3.60E-003
B. vansinderenii	-212064.17	-212061.75	4.84	8.91E-002	5.57E-002

Table S4. Values of the LRT that were applied to evaluate the presence of episodic positive selection in the sequences of the PFNA genes. The LRTs was performed in strict and relaxed conditions for the branches of the phylogenetic tree of bifidobacteria that passed test 1. The corresponding p-values are given. The result is statistically significant when $P < \alpha$. The ω values are specified for a group of sites of the foreground branch with a value of $\omega > 1$.

**foreground branch / clade**	**lnL value (A1 model)**	**lnL value (A model)**	**LRT value**	**P-value (strict conditions, α = 5.00E-004)**	**P-value (relaxed conditions, α = 5.00E-002)**	**ω value**
B. actinocoloniiforme	-211910.16	-211851.62	117.09	0	0	4.79
B. aesculapii	-212063.36	-212052.63	21.46	3.62E-006	3.76E-006	23.71
B. angulatum, *B. merycicum*	-211965.13	-211963.15	3.97	4.64E-002	2.51E-002	1.46
B. animalis subsp. *animalis*, *B. animalis* subsp. *lactis*	-212004.17	-212000.55	7.24	7.14E-003	4.19E-003	1.60
B. asteroides	-212022.65	-211985.51	74.27	0	0	7.22
B. biavatii	-212011.54	-211976.88	69.31	0	0	5.63
B. bifidum	-212062.27	-212047.66	29.22	6.00E-008	7.36E-008	21.64
B. bohemicum	-212059.73	-212044.93	29.61	5.00E-007	6.75E-008	11.60
B. bombi	-212047.48	-212027.85	39.26	0	0	6.89
B. boum	-212064.16	-212060.78	6.77	not tested under the strict conditions	5.22E-003	12.52
B. breve	-212050.48	-212050.48	0	1	1	1
B. catenulatum, *B. kashiwanohense*, *B. pseudocatenulatum*	-212062.61	-212060.94	3.36	not tested under the strict conditions	3.48E-002	3.37
B. choerinum	-212061.10	-212042.51	37.19	0	0	25.81
B. commune	-212057.18	-212047.15	20.04	7.57E-006	6.81E-006	7.48
B. coryneforme, *B. indicum*	-212037.27	-212022.76	29.01	7.00E-008	7.875E-008	4.51
B. cuniculi	-212053.51	-212007.55	91.91	0	0	19.09
B. eulemuris	-212020.79	-212015.17	11.24	7.99E-004	6.34E-004	3.57
B. gallicum	-212052.12	-212041.48	21.28	3.97E-006	3.83E-006	4.99
B. hapali	-211966.66	-211948.99	35.35	0	0	2.87
B. lemurum	-212021.01	-212016.75	8.53	3.50E-003	2.25E-003	3.64
B. myosotis	-212063.87	-212058.82	10.09	not tested under the strict conditions	1.06E-003	23.68
B. pseudolongum subsp. *globosum*	-212050.38	-211994.42	111.93	0	0	16.46
B. pseudolongum subsp. *pseudolongum*	-212064.06	-212059.50	9.12	not tested under the strict conditions	1.71E-003	22.29
B. ruminantium	-212063.64	-212059.56	8.17	not tested under the	2.62E-003	8.64

				strict conditions		
B. scardovii	-212029.70	-211991.13	77.14	0	0	8.27
B. stellenboschense	-212064.13	-212056.48	15.30	not tested under the strict conditions	7.74E-005	44.86
B. tissieri	-212064.06	-212058.77	10.57	not tested under the strict conditions	8.62E-004	14.09

Table S5. Candidate amino acid sites under episodic positive selection in various tested branches of the phylogenetic tree based on concatenated sequences of the PFNA genes (PP > 0.7). The sites with PP > 0.95 are marked with an asterisk. The most reliable candidates for positive selection verified with an additional approach are highlighted in black.

foreground branch / clade	*pkb2*	*fn3*	*aaa-atp*	*duf58*	*tgm*
B. actinocoloniiforme	32 78 84* 128 169 196 276 510 512 550	146 152 237 286 288 326 362 371 381 383* 397* 401 420 427 454 464 465 470 474 482 502 505 516 532 540 546 572 580 587 592 **603*** 618* **620** 623 634 636 649 656* 664 674* 677* 680 681 682* 689 707 727 732* 733 734 735* 751 753 776* 778 792* 796 800 805 815 824 832 845* 859 862 865 869* 884 893 896 899 910 917 926 **948** 951 955* 957 966 969 988* 1006 1015 1027 1034* 1039* 1040* 1043 1045 1054 1063 1065* 1076* 1086* 1096 1100 1109 1114 1120* 1121 1127* 1132 1141 1150 1156 1164* 1172* 1173* 1199 1201* 1209 1216 1219* 1233* 1234* 1245 1260 1291 1299 1301 1311 1325 1330 1343* 1346 1359 1403 1417* 1440 1466 1474 1481* 1507 1511 1530 1532* 1535 1578 1579* 1656 1719	153 172 183 198* 203 224 226 254 277 289* 309 310* 324 327 345 346 357 365 371* 379 400 411* 412 415 436 445	49 67 68* 77 80 81 101 110 117 131 143 153 159* 160 163 165 172* 183 189 190 198* 199* 227 237* 239 244* 259 268 274 **275** 285 286 297 299 338 355 367 388 390 391 393* 396* 397 406	84 107 118 120 139* 144 145 152 160 161 163 166 170* 181* 249 254 267* 270* 279* 298* 299 300* 301 302 308 316 318 323 329 330 340 349* 356 357 366* 384 397 400* 436 440 445 470 481 485 486* 546 554 557 568 590 623 627* 628* 629 632 635 636 637* 653 655 687 703 704 710 712 713 721 734 788
B. aesculapii		**258** 585 1509			134 **136**
B. asteroides	**57** 125 135* 256* 383	184 **223*** 421 457 463 472* 675 688 713 766 808 823 833 847 902 1091 1128 1151 **1199** 1287 1305 1339 1382 1522 1553	155 301 333 402*	100 130 131 132 158 198 258 262 266 **281** 288 349 364 370 371 398 400	140 204 211 294* 369 381 411 432 602 605 611 630 700 710
B. biavatii	**41** 74 85 116 117 131 197 239 **243** 249	137 218 290 306 407 426 448 529 638 666 712 738 778 817 888* 951 997 1055 1089* 1099 1196 1214 1277 1350 1439 1535 1552 1627 1725	199 219 269 271	**43** 45 **56** 69 78 99 106 113 123 139 163 **226** 296 342 353* 366 396 400	**128*** 130 137 162 186 191 276 283 309 323 371 421 496 505 549 581* 603 648 657 669 677 **678** 706 70
B. bifidum	120 **246**	**260** 315 355 1362 1589		**80**	90 **144** 171 606 716
B. bohemicum	231	**360** 535 549 659 690 1241 1501 1520	244 383	84 **90** 219 398	372 378 419 534 **575** 610
B. bombi	131 145 160 286	466 607 693* 903 1067 1281 **1311** 1334		112 129 199	76 82 118 175 196 230 247 413 416 428 450 621 646 685 729 739 743
B. choerinum	121* **130** **166**	316 377 706* 1649* 1650 1676		238* **395**	
B. commune	156 285 286	**177** 391 774 824 912 1189	274 326	164 423	86 223 464 703
B. coryneforme, *B. indicum*	132	143 358 587 870 899 1052 1108 1555	97 382	69 151 371	569 662
B. cuniculi	90*	**262** 319 415* 513 594 **598*** 778 781 792 **838*** 867 967 1192 1245 1259 1276 **1278*** 1363 1433 1497 1522 1523 1542 1573 1715*		309	106* 126 **269** 312 347 419 **421** 597 635 745
B. gallicum		470 528 534 542 601 782 800 950 1044 1094 1370 1423		63	177 270 314

		1465 1541 1622 1659			
B. hapali	84 86 130 204 233 249 293 339	3 8 18 145 166 237 268 274 284 305 314 316 423* 430 442 446 465 468 469 475 481 488 518 533 551 577 591 592 620 623 626 670 691 692 716 718 772 778 808 875 881 886 912 934* 936 971 979* 1008 1031 1118 1187 1189 1201 1271 1297 1330 1366 1390 1392 1421	56 142 144 150 239 274 295 302 341 342 346	107 117 121 138 161 168 195 197 203 206 232 242 243* 252 282 295 319 330 387* 401 421 440 445	50 71 74 78 92 99 118 146 203 220 261 262 297 299 301 313 341 342 459 483 504 559* 565 621 628 679 693 700 705 741
B. pseudolongum subsp. *globosum*	133* 168	146 186 191 241 324 548 635* 849 853 950* 969 1055 1076 1089* 1130 1145 1251* 1256 1297* 1340 1397* 1419* 1442 1464 1496 1501 1541 1577	176* 242 322 362	137 238 369*	115* 129 167 209* 272 276 315 350 366 454 458 536
B. scardovii	72 96 103 119 149 411 455	127 446 506 549 698 744 779 821 850 1005 1021 1053 1063 1075* 1079 1086 1194 1202 1221 1243 1285 1295 1401 1479	125 267* 393	78 107 199 200 268 373	70 92 93* 139 168 235 274* 357 358 561 582 589 593 596*
